# Differentiating Pond-Intensive, Paddy-Ecologically, and Free-Range Cultured Crayfish (*Procambarus clarkii*) Using Stable Isotope and Multi-Element Analysis Coupled with Chemometrics

**DOI:** 10.3390/foods13182947

**Published:** 2024-09-18

**Authors:** Zhenzhen Xia, Zhi Liu, Yan Liu, Wenwen Cui, Dan Zheng, Mingfang Tao, Youxiang Zhou, Xitian Peng

**Affiliations:** 1 Hubei Key Laboratory of Nutritional Quality and Safety of Agro Products, Institute of Agricultural Quality Standards and Testing Technology Research, Hubei Academy of Agricultural Science, Wuhan 430064, China; wenwencui.305@163.com (W.C.); zhengdan@hbaas.com (D.Z.); taomingfang@hbaas.com (M.T.); zhou_youxiang@aliyun.com (Y.Z.); pxitian@aliyun.com (X.P.); 2 College of Agriculture and Biotechnology, Hunan University of Humanities, Science and Technology, Loudi 417000, China; 3 College of Food Science and Engineering, Wuhan Polytechnic University, Wuhan 430023, China; liuyanwhpu@163.com

**Keywords:** crayfish, farming pattern, stable isotope, multi-element, PLS-DA, authentication

## Abstract

The farming pattern of crayfish significantly impacts their quality, safety, and nutrition. Typically, green and ecologically friendly products command higher economic value and market competitiveness. Consequently, intensive farming methods are frequently employed in an attempt to replace these environmentally friendly products, leading to potential instances of commercial fraud. In this study, stable isotope and multi-element analysis were utilized in conjunction with multivariate modeling to differentiate between pond-intensive, paddy-ecologically, and free-range cultured crayfish. The four stable isotope ratios of carbon, nitrogen, hydrogen, and oxygen (δ^13^C, δ^15^N, δ^2^H, δ^18^O) and 20 elements from 88 crayfish samples and their feeds were determined for variance analysis and correlation analysis. To identify and differentiate three different farming pattern crayfish, unsupervised methods such as hierarchical cluster analysis (HCA) and principal component analysis (PCA) were used, as well as supervised multivariate modeling, specifically partial least squares discriminant analysis (PLS-DA). The HCA and PCA exhibited limited effectiveness in classifying the farming pattern of crayfish, whereas the PLS-DA demonstrated a more robust performance with a predictive accuracy of 90.8%. Additionally, variables such as δ^13^C, δ^15^N, δ^2^H, Mn, and Co exhibited relatively higher contributions in the PLS-DA model, with a variable influence on projection (VIP) greater than 1. This study is the first attempt to use stable isotope and multi-element analysis to distinguish crayfish under three farming patterns. It holds promising potential as an effective strategy for crayfish authentication.

## 1. Introduction

Crayfish (*procambarus clarkii*) is a highly valued species in China that is known for its nutritional composition, including rich protein content, unsaturated fatty acids, and various trace elements [1]. In recent years, crayfish farming has rapidly expanded, making it one of the most extensively cultivated aquaculture products. By 2022, the production had reached 2,890,700 tons, placing crayfish as the fourth-largest freshwater aquacultural product in China [2]. According to estimates of the fishery administration of the Ministry of Agriculture and Rural Affairs, the crayfish industry’s total output value reached about CNY 458 billion in 2022. Currently, crayfish aquaculture is classified into four patterns, i.e., paddy-ecological, pond-intensive, lotus pond crayfish, and large water surface aquaculture [2,3]. Among these patterns, paddy-ecological and pond-intensive models dominate crayfish production, covering approximately 89.93% and 12.50% of the total farming areas, respectively [2].

The paddy-ecological pattern utilizes at least a 10% portion of the paddy field area for crayfish ditches, which is called the rice crayfish integrated and breeding model. Currently, the output of crayfish in the paddy-ecological breeding pattern accounts for 83% of the total annual yields [4]. The rice crayfish integrated breeding model, particularly rice crayfish co-culture, is considered a new type of green, eco-friendly, and sustainable pattern [5]. In the pattern, rice cultivation offers optimal growth conditions for crayfish. This not only increases the three-dimensional space available for crayfish development but also broadens their activity range. Rice crayfish cultured in the paddy are favored by consumers due to their better taste and higher nutritional value compared to pond crayfish cultured in an intensive pond [6]. In contrast, in the past decade, pond-intensive agriculture has been widely used to increase crayfish yields and economic benefits [7,8]. The production of pond crayfish accounts for 14.8% of the total crayfish production. In intensive ponds, a large number of crayfish is released, resulting in high crayfish farming densities. In order to prevent and control the occurrence of fish diseases and promote the growth of crayfish, a large amount of feed and veterinary drugs is used, which leads to safety issues with the quality of aquatic products [8,9]. Crayfish display an absolute competitive advantage in the local ecology because they are omnivorous, have fast growth, and are highly adaptable. In the wild, crayfish usually breed in any convenient pond, but wild crayfish have posed the risk of heavy metal contamination due to the crayfish’s food and ecological environment being uncontrollable. The average amounts of heavy metals are found to be higher in crayfish from uncultivated ponds than in cultivated ponds [10]. 

As consumers pay more attention to food quality, the demand for green and environmentally friendly aquaculture products has increased. Rice crayfish, as an ecologically cultured product, has received more consumer preference, resulting in greater sales volume and market value [11,12]. Because of benefit-seeking, immoral producers may counterfeit culture model labels and sell products illegally. China’s Food Safety Law stipulates that the labels and instructions of food must be true. Therefore, credible and operational techniques are required for tracing and determining the origin of products to ensure consumer interests and confidence. 

Food safety and authentication issues have become increasingly severe in recent years [13,14]. In China, there are strict regulations regarding the integrity of food information, covering aspects such as variety, origin, planting, processing, and sales, to ensure that consumers have confidence in the food they consume. So far, identification techniques of aquatic product origin, provenance, and farming patterns, such as high-performance liquid chromatography (HPLC), nuclear magnetic resonance (NMR) spectroscopy, near-infrared spectroscopy (NIR), capillary electrophoresis, based deoxyribonucleic acid (DNA-based) and proteomic methods, metabolism analysis, stable isotope analysis, multi-element analysis, etc., are used to discriminate the origin of aquaculture products [12,15,16,17]. However, the above analytical methods for the origin discrimination of aquatic products are emphasized; for example, HPLC and metabolism analysis are suitable for the separation of metabolite markers and capillary electrophoresis, while DNA-based and proteomic methods are usually applied for species identification. NMR is non-destructive in nature and has the ability to provide detailed information about the molecular structure of samples, making it of great importance in adulteration identification [18]. NIR techniques are extensively utilized in food traceability due to their non-destruction, rapid, and user-friendly features, but the method does not provide an interpretation of chemical tracers that are directly linked to their origin [18,19]. 

Among these methods, stable isotope and multi-element analysis are two techniques that have demonstrated potential in assisting other analytical technologies for aquaculture pattern authenticity and provenance [20,21]. A “natural fingerprint” representing trophic position, habitat or ecological conditions, and nutritional inputs is provided by stable isotopes. Generally, the isotope ratios of carbon and nitrogen (δ^13^C and δ^15^N) tend to be increase in consumers compared to their food sources, a phenomenon known as fractionation or trophic enrichment. The change in δ^13^C is about 0.5~1‰ substantially from primary producers to predators, while the increase in δ^15^N is approximately 2~3‰ for each trophic level. It is common knowledge that isotope ratios of hydrogen and oxygen (δ^2^H and δ^18^O) of aquatic products are closely linked to environmental water. Hence, it is sometimes not obvious to differentiate production patterns. Against this background, multi-element analysis as a supplementary measure is commonly applied for the traceability of aquatic product origins [20,22]. The trace elements are accumulated as the varying farming areas, patterns, and feed types. Appropriate trace elements may be selected and used for identifying the origin of aquatic products. Numerous studies have explored the utilization of trace elements and stable isotope analysis in identifying the farming patterns of aquatic products [23,24,25,26], such as the identification of wild and farmed Atlantic salmon, gilthead sea bream, Asian seabass, shrimps, and carp. The composition of elements and stable isotopes in aquatic animal products is directly affected by regional climatic conditions, biological environmental interactions, and biological metabolism. They record evidence of biological, chemical, and physical environmental processes. The differences in δ^13^C, δ^15^N, δ^2^H, and δ^18^O in different farming patterns were pointed out in the research by Zhao et al. [27]. Moreover, variations in the distribution of C3 and C4 plants across latitudes, coupled with the characteristic lower ^13^C/^12^C ratios of C3 plants compared to C4 plants, result in discernible differences in ^13^C/^12^C ratios within plant materials based on their geographical origins [28,29]. Variations in the ratios of ^15^N/^14^N are primarily influenced by the specific farming methods used in the area [29,30]. As carbon and nitrogen move from prey to predator within the food chain, the isotope ratios of ^13^C/^12^C and ^15^N/^14^N provide insight into the feeding interactions over time. Hence, it is feasible to use stable isotope and multi-element analysis for differentiating crayfish farming patterns. 

However, the remarkable thing is that crayfish farming pattern traceability research has limitations that need to be addressed. (1) Crayfish cultured in paddy-ecological, pond-intensive, and free-range environments share a common water source, which similarity restricts the applicability of the method to only stable isotope analysis. (2) The impact of crayfish size variation on stable isotopes and multi-element analysis cannot be ignored, and sampling should involve similar-sized crayfish to minimize size-related biases. (3) Various multivariate modeling techniques, such as unsupervised methods like hierarchical cluster analysis (HCA) and principal component analysis (PCA), as well as supervised analysis like partial least squares discriminant analysis (PLS-DA), should be evaluated in conjunction with variable screening to enhance the accuracy of discrimination.

Currently, there are studies on the use of stable isotopes combined with multi-element methods for the study of fish farming methods and geographical origins [13,26,28], but research on the classification of crayfish farming patterns has not been found. This research seeks to assess the isotopic and multi-element profiles of crayfish, environmental samples, and feeds; investigate the significance and correlations between crayfish sourced from various aquaculture methods and environments; and develop a multivariate statistical analysis model utilizing stable isotopic and multi-element data to differentiate farming patterns. This study aims to offer a methodology for tracing the farming method of crayfish in China, potentially serving as a supplementary tool for governmental oversight of crayfish commerce and verifying the authenticity of crayfish labeling.

## 2. Materials and Methods

### 2.1. Sampling Collection

**Figure 1:**
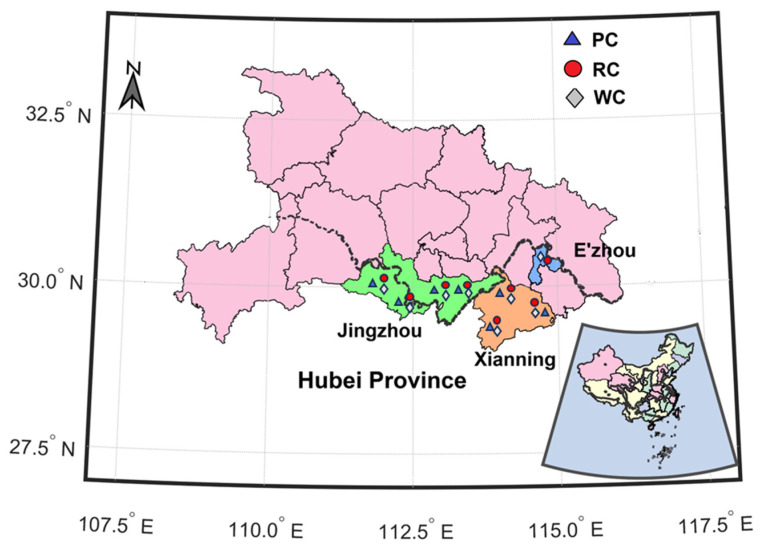
Map of crayfish sampling sites from different farming patterns in Hubei province. PC, RC, and WC represent pond crayfish, rice crayfish and wild crayfish, respectively.

In the study, all samples were obtained directly from local farms in the primary cultivation regions. A combined total of 88 crayfish samples within rice crayfish co-culture, intensive ponds, and wild rivers were collected in Jingzhou, Xianning, and E’zhou cities, Hubei province, China, from April to July 2021 (shown in Figure 1). These crayfish samples were divided into three groups based on culture mode, including *N* = 26 samples from intensive ponds, *N* = 38 samples from rice crayfish co-culture, and *N* = 24 samples from wild rivers. The sampling information is listed in Table S1. To reduce the influence of body weight, all sampled crayfish weighed approximately 25–40 g each. Each sample consisted of a minimum of 40 crayfish to ensure representation and eliminate any potential effect linked to variety or individual differences.

- Rice crayfish inhabit paddy fields and annular ditches on the periphery of rice fields. The combined cultivation area measured 80 m by 40 m, with the rice planting section occupying 66% of the total area. This rice field was enclosed by a trench that was 5 m wide and 1.5 m deep. Crayfish were captured using metal ground nets placed in four corners of the ring trench according to four-point sampling. Pond crayfish were cultivated in an intensive pond measuring 70 m in length, 35 m in width, and 2 m in depth. The collection process employed a five-point sampling method, which included the four corners and the center of the pond. The wild river crayfish, which occur in free-range waters naturally, were collected from wild rivers with an S-type sampling method along the river. The different sampling methods refer to the agricultural industry standards of the People’s Republic of China, NY/T 398-2000 [31] and NY/T 789-2004 [32]. All crayfish samples were added to ice and immediately transported to the research facilities. Once they reached the laboratory, live crayfish were chosen and rinsed with Milli-Q water to remove contamination on the carapace before sample preparation for analysis.
- The ecological environments of the three selected crayfish farming patterns, along with their respective feeding amounts, show some differences. These patterns include wild crayfish with no feeding, rice-field crayfish with less feeding, and pond crayfish with complete feeding. The compound feed was made of specialized granular feed from ingredients such as fish meat, tofu dregs, corn flour, and mixed fish meal. Crayfish are most active in feeding during the period from dusk to dawn; therefore, the crayfish were fed twice a day: once in the morning and once in the evening. To promote the growth of the crayfish, cooked soybeans were also fed in conjunction with commercial feed. Thus, two commercial feeds and two beans that came from different crayfish farms were collected.
- Aquatic plants are essential for crayfish to grow, which not only were necessary food for the crayfish but also provided the space for the crayfish to crawl. Two kinds of common aquatic plants, Elodea Canadensis Michx and Hydrilla verticillate, were collected, and the surface soil was washed clean with Milli-Q water.
- The sediments in the ring trench of the rice crayfish co-culture, intensive pond, and wild river were collected using different sampling methods, including the four-point method for the ring trench. Sediment samples were obtained from the top 10 cm of the substrate at various locations. A total of twenty-three sediment samples, each weighing around 1 kg, were gathered from the rice crayfish co-culture, intensive pond, and natural river environments utilizing a stainless-steel box corer and soil collector.

### 2.2. Sampling Pretreatment

All crayfish samples were frozen in accordance with the National Standard of China: Guidelines for Ethical Review of Laboratory Animal Welfare (GB/T 35892-2018) [33] to ensure adherence to pertinent guidelines and regulations of the global scientific community.

- Crayfish tail meat preparation: The tail meat of crayfish was obtained by stripping the crayfish’s shell and head, and at least 100 g of crayfish for each sample were collected. The tail meat was prepared into a puree using a blender, and then all crayfish meat was placed in clean Petri dishes and lyophilized at −80 °C for 72 h. The dried meat was pulverized by a mixer mill. Crayfish meat powder underwent a solvent extraction process to eliminate lipids following the methodology outlined by Schlechtriem, Focken, and Becker in 2003 [34].
- Feed sample preparation: The commercial feeds and soybeans were dried at 60 °C for 12 h using a draught drying cabinet. The dried samples were homogenized and ground into powder.
- Aquatic plant preparation: The root of the aquatic plant was cut off and the leaf was cleaned with deionized water. Subsequently, the aquatic plant was placed on the plate and also dried at 60 °C for 12 h using a draught drying cabinet. The fine powder (particle size < 80 mesh) was prepared by a milling system.
- Sediment preparation: The sediments were dried naturally in the chamber, and then the sediments were broken and ground into a fine powder with an 80-mesh sieve.

All samples were stored frozen at −20 °C before isotope and elemental analysis.

### 2.3. Stable Isotope Ratio Analysis

The stable isotope ratios of δ^13^C, δ^15^N, δ^2^H, and δ^18^O were measured through analysis conducted with a Flash EA 2000 elemental analyzer connected in continuous flow to a Delta V Plus mass spectrometer (Thermo Fisher Scientific^TM^, New York, NY, USA). To analyze δ^13^C and δ^15^N, roughly 1 mg of the prepared sample was weighed in triplicate. The weighted sample was carefully put into a tin capsule for isotope analysis. The sample’s carbon and nitrogen were transformed into CO_2_ and N_2_, respectively. These gases were then analyzed by an Isoprime 100 mass spectrometer (Isoprime, Manchester, UK), following purification through an adsorption column. The helium carrier gas was flowing at a rate of 230 mL/min, with the combustion furnace set at 1150 °C and the reduction furnace at 850 °C. Additionally, the helium flow rate into the mass spectrometer was 100 mL/min. To analyze δ^2^H and δ^18^O, around 0.5 mg of the sample was weighed and processed in a similar manner as previously. The sample’s hydrogen and oxygen were transformed into H_2_ and CO, and then examined using a Delta V Advantage mass spectrometer from Thermo Fisher Scientific™ (New York, NY, USA). The helium flow rate was 200 mL/min, the temperature of the combustion tube was 1400 °C, and the helium flow rate entering the mass spectrometer was 110 mL/min. The stable isotope ratio was determined using Equation (1) [35]:

*δ* (‰) = [(*R_sample_*/*R_standard_*) − 1] × 1000 (1)

where *δ* represents either δ^13^C, δ^15^N, δ^2^H, or δ^18^O. The variables *R_sample_* and *R_standard_* represent the relative abundance of isotopes in samples and reference materials, specifically, the ratio of “heavy” to “light” isotopes such as ^13^C/^12^C, ^15^N/^14^N, ^2^H/^1^H, and ^18^O/^16^O.

In this research, reference standards obtained from the International Atomic Energy Agency in Vienna, Austria, were utilized as quality control samples for the for multi-point calibration of isotopic ratios. Specifically, these references included IAEA-CH6 (sucrose, δ^13^C = −10.4‰), IAEA-600 (caffeine, δ^13^C = −27.8‰, δ^15^N = +1.0‰), and B2157 (wheat flour, δ^13^C = −27.2‰, δ^15^N = +2.8‰) for δ^13^C; IAEA-N1 (ammonium sulfate, δ^15^N = +0.4‰) for δ^15^N; VSMOW (Vienna standard mean ocean water, δ^18^O = 0‰ and δ^2^H = 0‰) and SLAP (standard light Antarctic precipitation, δ^2^H = −428.0‰) for δ^2^H; and IAEA-601 (benzoic acid, δ^18^Ovsmow = +23.3‰) and IAEA-602 (benzoic acid, δ^18^Ovsmow = +71.4‰) for δ^18^O. The level of instrumental accuracy and consistency, as indicated by relative standard deviations (RSDs), was observed to be below ±0.2‰, ±0.2‰, ±1.5‰, and ±0.3‰ for δ^13^C, δ^15^N, δ^2^H, and δ^18^O, respectively. The carrier gas utilized in isotopic analysis was high-purity helium gas with a concentration exceeding 99.99%, and additionally, reference gases such as CO_2_, N_2_, H_2_, and CO were sourced from Jingong Special Gas Co., Ltd. in Changsha, China.

### 2.4. Multi-Element Analysis

Precisely 0.5 g of crayfish powder was measured and placed in a PTFE digestion vessel, followed by the addition of 5 mL of nitric acid (65% concentration, weight/weight, MOS grade), and left undisturbed for about 24 h. Prior to microwave digestion, 2 mL of hydrogen peroxide (30% concentration, weight/weight, MOS grade) were introduced. The samples underwent digestion for one hour utilizing the microwave digestion system (MARS 6, CEM Corp, Matthews, NC, USA). The resulting digestion solution was cooled to around 20 degrees Celsius, then transferred to a 50 mL volumetric flask and adjusted to a final volume of 50 mL with 2% nitric acid (volume/volume).

The concentration of multiple elements (B, Na, Mg, Al, K, Ca, Ti, V, Cr, Mn, Fe, Co, Ni, Cu, Zn, Mo, Cd, and Pb) was analyzed using ICAP-6000 inductively coupled plasma mass spectrometry (ICP-MS) (Thermo Fisher Scientific in Shanghai, China). The most effective operational parameters for ICPMS included a radio frequency power of 1550 W, utilization of a kinetic energy discrimination model with a helium flow rate of 4.35 mL/min, employment of a quartz glass concentric nebulizer, a sampling duration of 0.1 s, and the repetition of the process twice. Internal standards consisting of 10 μg/L of Rh, Re, In, and Ge (obtained from a single element standard by Inorganic Ventures, Christiansburg, VA, USA) were employed to mitigate matrix effects and instrument variations. Quantitative analysis was conducted using the external standard method, with determination coefficients of standard curves exceeding 0.99. Results were reported as the average of two test outcomes. A reagent blank was analyzed with eleven replicates following the same protocol as the crayfish sample to determine the limit of detection [36].

### 2.5. Chemometrics

Chemometric methods, including HCA [37,38,39], PCA [37], and PLS-DA [40,41], were applied for discriminating the crayfish farming patterns. HCA is a method used for data reduction that organizes data into subgroups to facilitate analysis, enabling users to observe the similarities among the samples being studied. In the HCA approach, each data point is initially considered as an individual cluster. The method then computes the Euclidean distances across all clusters and employs the minimum distance method to pinpoint the two clusters with the smallest distance between them. Proceeding from the calculated distances, the two closest clusters are singled out and fused into a unified cluster. This action creates a subsequent level of clustering. This cycle of identification and merging continues until the desired number of clusters is achieved, all the while constructing a dendrogram that illustrates the progression of the clustering. In the process of applying PCA, it is standard practice to determine the covariance matrix, which quantifies the linear correlations among variables. The objective of PCA is to identify the axes along which the variance is maximized, a task typically accomplished through the computation of the covariance matrix’s eigenvalues and eigenvectors. PCA extracts the most important principal components from a large number of variables to simplify the data structure and reveal the relationships between variables. Both HCA and PCA are unsupervised classification methods. PLS-DA is a supervised classification method and can perform feature selection and dimensionality reduction simultaneously, while considering the relationship between multiple independent variables and the dependent variable.

The multi-element and stable isotope data from the 88 crayfish samples obtained in the experiment were normalized. Following the normalization, the data had an average value of 0 and a standard deviation of 1. The normalized data were then used for subsequent HCA, PCA, and PLS-DA analyses. For PCA, the scores of the first two principal components were selected for investigation. For PLS-DA, the sample sets from different farming patterns were divided into two sets randomly: a training set and a test set, consisting of 60 and 28 samples, respectively. Leave-one-out cross-validation (LOOCV) was used to calibrate the model using the samples of the training set. The test set was used to validate the discriminant model. The accuracy was the criteria of the model, which was calculated by dividing the number of correctly classified samples by the total number of samples, and then multiplying by 100 to obtain the accuracy rate in percentage form, as in Equation (2).

(2) $$Accuracy=\frac{TP+TN}{TP+FN+FP+TN}\times 100\%$$

where true positive (TP) indicates the number of positive samples that were correctly predicted, true negative (TN) indicates the number of negative samples that were correctly predicted, false positive (FP) indicates the number of negative samples that were incorrectly predicted as positive, and false negative (FN) indicates the number of positive samples that were incorrectly predicted as negative. 

In this study, analysis of variance (ANOVA) comparisons and correlation analysis of variables were performed in MATLAB software (version 2019a). These chemometric methods were also processed by SIMCA-P and MATLAB for modeling crayfish samples. All figures were drawn by Origin and SIMCA-P.

## 3. Results and Discussion

### 3.1. The Variances in C, N, H, and O Isotope Ratio Values in Crayfish between Different Patterns

The average values, deviations, and ANOVA comparison outcomes for stable isotope ratios of δ^13^C, δ^15^N, δ^2^H, and δ^18^O in the crayfish were compiled through stable isotope analysis and are shown in Table 1.

**Figure 2:**
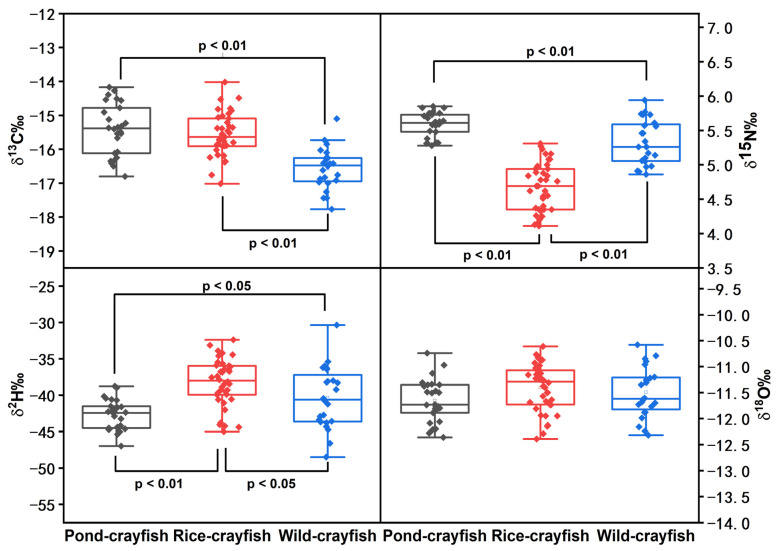
The ANOVA boxplots depict stable isotope ratios (δ^13^C, δ^15^N, δ^2^H, and δ^18^O) of crayfish from different farming patterns.

The results of the carbon stable isotope ratio for crayfish show that pond crayfish had the highest mean δ^13^C value of −15.42 ± 0.76‰, and rice crayfish and wild crayfish had lower δ^13^C, which were −15.58 ± 0.64‰ and −16.57 ± 0.6‰, respectively. According to ANOVA, δ^13^C the wild crayfish group had a significant difference compared to the other two groups (*p* < 0.01, shown in Figure 2). Due to the carbon-stable isotope ratio in crayfish being closely related to feed, the main diet of crayfish was considered the reference for δ^13^C. The crayfish’s main diet included commercial feed, aquatic plants, and beans, and the mean value of δ^13^C was analyzed, with a result of −27.78 ± 0.44‰, −28.63 ± 0.45‰, and −27.83 ± 0.25‰, respectively. Compared with the crayfish’s δ^13^C, the values of the crayfish’s diet were more positive. In addition, the mean δ^13^C value in pond crayfish was the highest, rice crayfish’s δ^13^C value was lower, and the wild crayfish’s value was the lowest. This might be because compared with rice crayfish and wild crayfish, pond crayfish were mainly fed the largest portion of feeds containing C4 terrestrial plants (e.g., maize). 

From highest to smallest, the average δ^15^N of crayfish were 5.59 ± 0.18‰, 5.33 ± 0.32‰, and 4.69 ± 0.35‰, respectively. According to ANOVA, the δ^15^N in three groups of crayfish had significant differences with each other (*p* < 0.01, shown in Figure 2). Pond crayfish exhibited the most positive δ^15^N, while wild crayfish had a more positive δ^15^N than rice crayfish. Usually, the nitrogen-stable isotope ratio is associated with the agricultural method employed in raising farm animals. Rice crayfish were cultured using shallow water in a paddy field, and the rice’s roots absorb nitrogen from the environment for growing. As rice plants absorb nitrogen from the environment for their growth, this resulted in the rice crayfish having relatively lower δ^15^N values compared to the other two groups.

The stable isotopes of δ^2^H and δ^18^O in aquatic organisms were found to be primarily influenced by the cultured water, with less impact from their dietary intake. The δ^2^H values of rice crayfish, pond crayfish, and wild crayfish were −38.29 ± 3.28‰, −42.74 ± 2.08‰, and −40.39 ± 4.22‰, respectively. The mean δ^2^H was significantly different in rice crayfish, pond crayfish, and wild crayfish (*p* < 0.05, shown in Figure 2), with the rice crayfish and pond crayfish groups showing extremely significant differences (*p* < 0.01, shown in Figure 2). The report shows that the δ^2^H values of crayfish are related to cultured water and that the water’s hydrogen isotope ratio is influenced by climate and physiological processes such as photosynthesis. Due to different photosynthesis in paddy fields, ponds, and rivers, significant differences were observed in different culture methods. For the δ^18^O values of crayfish, there were insignificant differences in different groups (*p* > 0.05). Therefore, δ^18^O in crayfish was stable among different culture methods. 

### 3.2. The Variances in Multi-Elemental Concentration in Crayfish between Different Patterns

A few multiple elements below the detection limit were excluded, and the mean and deviation of the remaining elements are listed in Table 2. These elements fall into three distinct groups based on their content values. The first group consists of elements with average contents exceeding 100 mg kg^−1^. The second group includes elements such as Mn, Fe, Cu, and Zn, with contents ranging from 13 to 50 mg kg^−1^. The remaining elements fall into the third group, with contents lower than 1 mg kg^−1^. As per the guidelines outlined in the national standard GB 2762-2022 [42], which sets the Food Safety Limits of Contaminants in Food, the permissible limits for Pb, As, and Cd are less than 0.5 mg kg^−1^, while for Cr it is less than 2.0 mg kg^−1^. The levels of Pb, As, Cd, and Cr in the various groups of crayfish analyzed conform to the stipulations of the national standard. 

The elemental content in crayfish is primarily influenced by the elements in their feed and the soil in their activity areas. The contents of 11 elements, including B, Sc, V, Cr, Co, Ni, Mo, Sb, Te, Pb, and Bi, showed very little variation among the three farming methods, with the mean differences all being less than 0.05 mg/kg. For the elements of Be, Fe, Cu, and As, the highest contents in wild crayfish were 179.80 ± 92.25 mg/kg, 49.61 ± 13.15 mg/kg, 16.03 ± 6.25 mg/kg, and 0.39 ± 0.14 mg/kg, respectively. In contrast, pond crayfish and rice crayfish are more intensively managed, and the soil environment and food sources in wild rivers are more complex, which may lead to higher contents of these elements in wild crayfish compared to pond crayfish and rice crayfish. During the process of rice cultivation, fertilizers containing Zn are sprayed to promote rice production and increase yields, resulting in the highest Zn content in rice crayfish. By ANOVA, the comparison results of each element among rice crayfish, pond crayfish, and wild crayfish showed that there was no significant difference between the elemental characteristics of crayfish in the different models examined (*p* > 0.05).

### 3.3. Isotopic and Multi-Elemental Relationships among Crayfish, Aquatic Plants, and Feed

**Figure 3:**
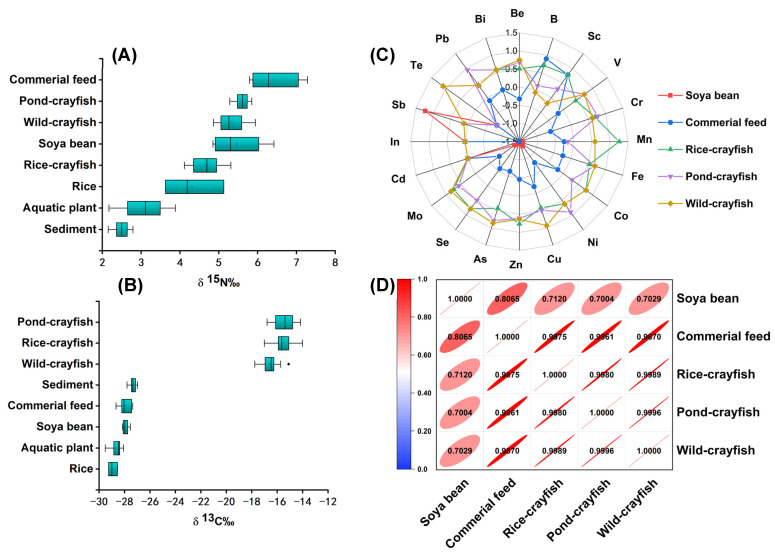
Boxplots of δ^13^C (**A**) and δ^15^N (**B**) of crayfish from different farming patterns, environment samples (e.g., sediment, aquatic plants, and rice), and feed samples (e.g., commercial feed and soya bean); (**C**) radar plot of the mean values of 20 elemental contents in crayfish and feeds; (**D**) correlation heat map of crayfish and feeds.

It is well known that differences in isotope abundance in organisms are closely related to their food sources and environment [43]. The δ^13^C and δ^15^N isotopic signatures of crayfish’s dietary resources (e.g., commercial feed and soya bean) and environmental samples (e.g., rice, aquatic plants, and sediment) are shown in Figure 3A,B, and the mean values and sample information are listed in Table S2. The δ^13^C ratio values and standard deviations of rice, aquatic plants, soya bean, commercial feed, and sediment were −28.97 ± 0.35‰, −28.63 ± 0.45‰, −27.96 ± 0.50‰, and 27.33 ± 0.22‰, respectively, appearing to range from minor to changing greatly. Comparing the feed resources of crayfish, the δ^13^C ratio value of the commercial feed was higher than that of soybean because the commercial feed may contain corn, which is a C4 model plant that has a higher δ^13^C ratio value than the C3 model plant (e.g., soya bean). In environmental samples, the sediment had the highest δ^13^C ratio value compared to rice and aquatic plants because sediment contains large amounts of inorganic carbon, organic carbon, and carbonates. 

From small to large, the δ^15^N ratio values and standard deviations of sediment, aquatic plants, rice, soya bean, and commercial feed were 2.49 ± 0.18‰, 3.07 ± 0.58‰, 4.31 ± 0.76‰, 5.15 ± 0.42‰, and 6.43 ± 0.63‰, respectively. It was found that the δ^15^N ratio of feed resources was higher than that of environmental samples because there was a large amount of animal protein and plant protein in feed samples. Both the δ^13^C and δ^15^N ratio values of aquatic plants were considerably lower than those of the other environment samples. The δ^15^N ratio of commercial feed was higher than that of soybean due to its inclusion of animal protein. Additionally, the δ^13^C and δ^15^N ratio values of aquatic plants were significantly lower compared to those of other environmental samples.

Hydrogen and oxygen isotopes primarily change through the evaporation and precipitation of seawater, and fractionation occurs during this process [44] (Dansgaard, 1964). Generally, the δ^2^H and δ^18^O values in precipitation become more negative with increasing latitude, altitude, and distance inland, and decreasing temperature. The δ^2^H and δ^18^O values in crayfish tissue are mainly influenced by the culture water, and the δ^2^H and δ^18^O values in the water body can vary with different seasons, precipitation, altitude, and latitude of survival [45]. Since the three farming patterns are all distributed in the Jianghan Plain, the differences in season, rainfall, altitude, and latitude are not significant. Therefore, there is no discussion of the influence of δ^2^H and δ^18^O isotopes on environmental samples (feed and aquatic plants).

The signatures of aquatic plants and feed are shown in Figure 3C, which shows the data processed by standardization. Due to the obvious differences in element content among different samples, the data of different orders of magnitude were transformed into uniformly measured data through standardization for improving comparability. Multi-element contents of feed resources are listed in Table S3. The trend of the content of each element is consistent with that in crayfish samples. The contents of Be, Mn, Fe, Cu, and Zn in the feed were in the range of 2.08–107.26 mg kg^−1^, and the contents of other elements were all less than 1.00 mg kg^−1^.

In the ecosystem, crayfish have the highest nutrient levels compared to aquatic plants, soybeans, feed-source organisms, and sediment organisms. Through the enrichment of the food chain, the stable isotope ratio and multi-element contents of samples with high nutrient levels are higher than those with low nutrient levels. This was also confirmed by comparing isotopic and elemental signatures of environmental samples from different models of crayfish and feed samples. In Figure 3A, the δ^13^C ratio values of crayfish from different patterns were much higher than the feed and environment samples. The δ^15^N ratio values of crayfish were higher than those of environmental samples, as shown in Figure 3B. The δ^15^N ratio value of commercial feed was higher than that of crayfish, while soya bean’s δ^15^N ratio value was higher than that of rice crayfish but lower than that of pond crayfish and wild crayfish. In the radar plot in Figure 3C, it can be seen that most element contents of crayfish were higher than in feed samples, except for Sb.

Liner correlation analysis of different samples, including feed and crayfish, was analyzed, and the regression results are illustrated in Figure 3D. The correlation of different samples (e.g., soya bean, commercial feed, rice crayfish, pond crayfish, and wild crayfish) exhibits a strong positive relation, with correlation coefficients in the range of 0.9861–0.9875 between crayfish and commercial feed and correlation coefficients of about 0.70 between crayfish and soya bean. The result show that commercial feed could be the main diet resource of crayfish, and crayfish from different farming patterns had litter differences in the isotopic and multi-elemental signatures. The correlation coefficients among rice crayfish, pond crayfish, and wild crayfish were above 0.998, which indicates that the isotopic and multi-elemental signatures had similarities among crayfish from different farming patterns. In this context, there were no obvious characteristic variables that identified crayfish from different farming modes; consequently, the multivariate statistics method needs to be utilized.

### 3.4. Identification of Farming Patterns of Crayfish by HCA and PCA

**Figure 4:**
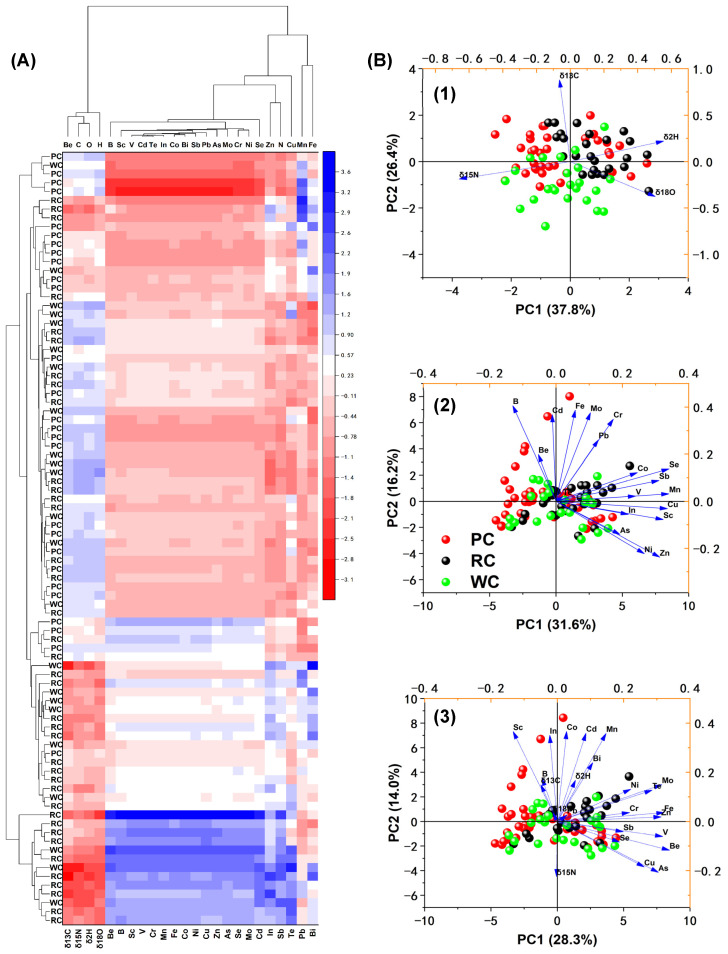
(**A**) Hierarchical clustering of different crayfish farming patterns and correlations of stable isotopic and multi-element data of crayfish with their farming patterns; (**B**) unsupervised PCA classification results of pond crayfish (PC), rice crayfish (RC), and wild crayfish (WC) scatter plots of the first two principal scores (PC1 and PC2) and corresponding variable loadings (**1**) only stable isotopic data used and (**2**) only multi-element data used; (**3**) both stable isotopic and multi-element data.

In the present investigation, HCA was employed to analyze stable isotope ratios and multi-elemental contents, as illustrated in Figure 4A. This analysis facilitated the differentiation of sample groups based on various farming patterns and provided insights into the associations between isotopic and elemental variables with crayfish samples.

In Figure 4A, the heatmap exhibits the relationships between samples and variables, which were constructed using normalized data of stable isotopes and multiple elements. The color bar indicates that the relative content of variables varied from −3.12 to 3.58, with red signifying a relatively high content after normalization and blue indicating a relatively low content. The dendrograms on the left and at the top represent the correlation between samples and between variables, respectively. In the dendrogram, samples or variables that are closer together indicate a higher correlation or similarity between them. Analysis of the heatmap revealed that 24 variables exhibited notable differences in expression across all crayfish farming patterns. Furthermore, the dendrogram for variables demonstrates the segregation of these 24 variables into distinct groups, e.g., a group of Be, δ^13^C, δ^18^O, and δ^2^H; a group of B, Sc, V, Cd, Te, In, Co, Bi, Sb, Pb, As, Mo, Cr, Ni and Se, Zn, and δ^15^N and Cu; and a group of Mn and Fe. The variables in the same group are highly correlated, and the variables in different groups are significantly different, so the variables present in distinct groups could serve as optimal indicators for classifying crayfish patterns. On the other hand, the dendrogram of crayfish samples indicates that rice crayfish were relatively well separated, whereas there were still heavy interferences for other patterns. For instance, In the left dendrogram, each sample was initially regarded as a separate group, and during the clustering process by HCA, similar samples were progressively merged together until all samples were combined into one large group. In the first level, two clusters formed. Cluster 1 refers to the clustering samples included in the lower node of the first level, while cluster 2 refers to the clustering samples included in the upper node of the first level. In cluster 1, rice crayfish were gathered as a group, but three wild crayfish became mixed up in it. In cluster 2, three pattern crayfish were mixed in and more subgroups were generated, in which most rice crayfish and pond crayfish gathered in a group, but wild crayfish became easily confused with the other pattern crayfish. As a result, the classification result of crayfish in different production patterns by HCA is still unsatisfactory. 

Next, PCA was performed to further analyze and confirm the separation among pond crayfish, rice-field crayfish, and wild crayfish, which were constructed using normalized data of stable isotopes and multiple elements. In Figure 4B, the differently colored circle symbols are the scores of different farming pattern crayfish, while the blue arrows represent the loading projection onto the same multivariate space of principal components and scores. Specifically, Figure 4B((1)–(3)) shows the PCA results using the data of stable isotopes, multiple elements, and a mixture, respectively. When stable isotope data were used, the PCA displayed a tendency of sample separation from various farming patterns. There was a clear separation trend between pond crayfish (red circle symbols) and rice crayfish (black circle symbols), but wild crayfish (green circle symbols) had some overlap with pond crayfish and rice crayfish. The vectors in the figure represent the loadings, which show the weighting of each variable. δ^13^C mainly contributed to PC2 to differentiate pond crayfish, rice crayfish, and wild crayfish, while δ^15^N, δ^2^H, and δ^18^O contributed to PC1 to differentiate crayfish. When multi-element data were used, the PCA showed that crayfish samples from different farming patterns were mixed (Figure 4B(2)). However, the use of mixed data did not improve the overlap between the different groups of samples (Figure 4B(3)). Comparing the scores of different data, the scores of the first two principal components were 64.2%, 49.3%, and 42.3%, respectively, and showed a gradual decrease. The results of the PCA did not improve the ability to extract information with an increasing amount of data. PCA is an unsupervised method, and generally, the first principal component does not separate well because it contains common information, which can be understood as the direction of minimum variance. PCA determines principal components along the direction of minimum variance, which may not necessarily reflect the differences between samples.

Therefore, due to the challenges encountered with unsupervised classification modeling approaches like HCA and PCA for crayfish production model traceability, it is recommended to explore supervised discriminant modeling methods to enhance the analytical performance. 

### 3.5. Discrimination of Farming Patterns of Crayfish by PLS-DA

**Figure 5:**
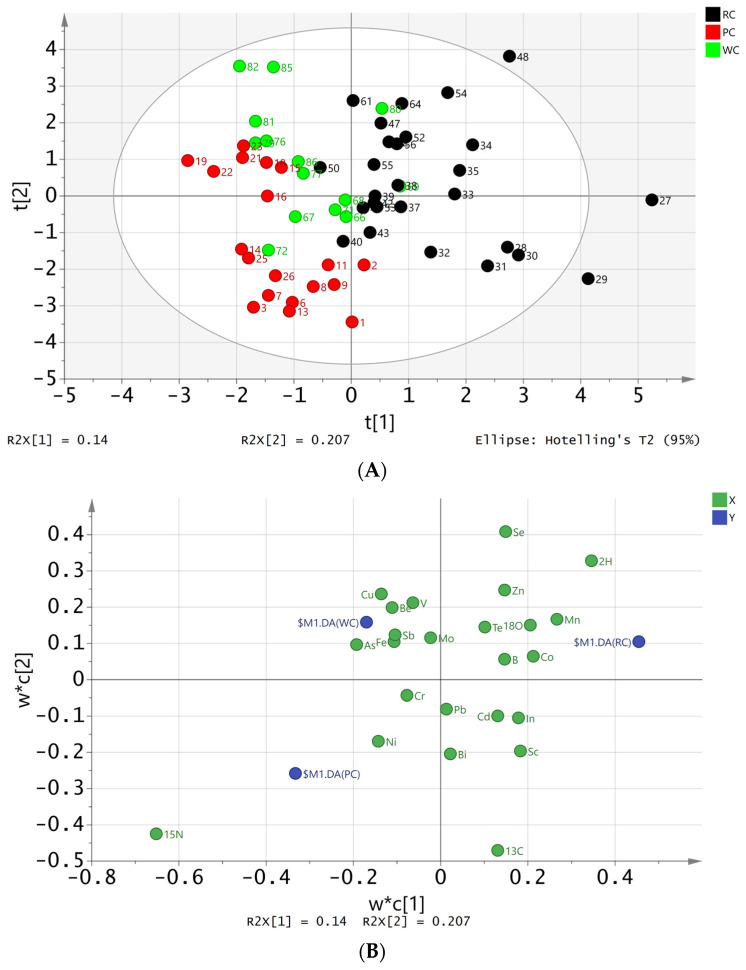

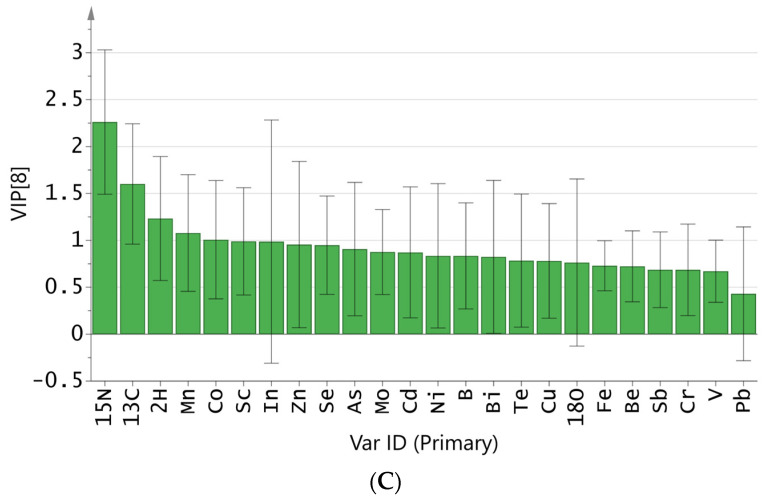
PLS-DA model using 4 stable isotopes and 20 elements from crayfish to differentiate pond crayfish (PC), rice crayfish (RC), and wild crayfish (WC): (**A**) score plot of samples; (**B**) loading plot of variables, with X standing for 24 variables and Y standing for three farming patterns; (**C**) VIP plot of variables.

To achieve a more accurate discrimination of farming patterns, a classical supervised discrimination method, PLS-DA, was employed. The sample sets from different farming patterns were divided into two sets: a training set and a test set, consisting of 60 and 28 samples, respectively. The accuracy rate, which represents the ratio of correctly predicted results to the total number of samples, was used to evaluate the prediction results. The predicted results of the test set are presented in Figure 5 and Table 3.

A supervised PLS-DA model was employed to cluster the crayfish into three distinct regions in a score scatter plot (Figure 5A). The symbols in red, black, and green represent rice crayfish, pond crayfish, and wild crayfish, respectively, from the training set. In the figure, it can be observed that most samples of the three types of crayfish could be separated, with a classification trend of rice crayfish being mainly distributed on the right side of the figure. Pond crayfish and wild crayfish were distributed on the left side, with pond crayfish mostly in the lower left corner and wild river crayfish in the upper left corner. There is some overlap between wild river crayfish and both rice crayfish and pond crayfish, but there is no overlap between rice crayfish and pond crayfish, indicating that the classification of pond crayfish and rice crayfish was more effective. The loading plot (Figure 5B) displays the weighting of each variable. δ^15^N, δ^2^H, Mn, and Co were found to be the main contributors to LV1, which differentiated rice crayfish from pond crayfish and wild crayfish. On the other hand, δ^13^C, δ^15^N, Se, Zn, Cu, Sr, and δ^2^H were the main contributors to LV2, which differentiated pond crayfish from wild crayfish. The variable influence on projection (VIP) plot of variables (Figure 5C) indicates that δ^15^N, δ^13^C, δ^2^H, Mn, and Co were the most important variables for classification (with VIP > 1). These results suggest that both isotopic and elemental variables from crayfish reflect the necessary ecosystem information required to differentiate pond-intensive, paddy-ecologically, and free-range cultured crayfish.

Table 3 shows that using PLS-DA, the predictive accuracy of pond crayfish, rice crayfish, and wild crayfish was 89.3%, 96.4%, and 85.7%, respectively. To assess the overall effectiveness of the prediction model, the total accuracy was calculated by adding the number of correctly predicted rice crayfish, pond crayfish, and wild crayfish, and then dividing by the total number of samples. The total predictive accuracy was found to be 90.8%. In summary, the PLS-DA models for pond crayfish, rice crayfish, and wild crayfish showed good discrimination performance. Therefore, the combination of four isotope and multi-element indexes provides a good strategy for the discrimination of crayfish from different farming patterns with PLS-DA.

## 4. Conclusions

In this study, significant differences were observed in the stable isotopes of δ^13^C, δ^15^N, and δ^2^H in crayfish from different farming methods. These variations can primarily be attributed to the application of farming patterns. Specifically, the mean δ^13^C value of pond crayfish was found to be most positive due to the inclusion of C4 plants in their feed, while the δ^15^N of rice crayfish was lowest due to the absorption of environmental nitrogen by rice roots. The δ^2^H values of crayfish are related to the culture water, and the three different culture water modes contain different algae and microorganisms, resulting in differences in the water. However, no significant difference was observed in the 20 elements analyzed, suggesting that multi-element analysis alone is not effective in differentiating crayfish farming methods. In order to distinguish different farming patterns, this study constructed HCA, PCA, and PLS-DA models using a combination of stable isotopes and multi-element data. The results showed that HCA and PCA are not suitable for distinguishing crayfish under different farming methods, but PLS-DA showed good performance. The predictive accuracy for pond crayfish, rice crayfish, and wild crayfish was 89.3%, 96.4%, and 85.7%, respectively, with an overall predictive accuracy of 90.8%. The variables that significantly contributed to the PLS-DA model included δ^13^C, δ^15^N, δ^2^H, Mn, and Co. Overall, this study established a simple, reliable, and adaptable technique for distinguishing between pond crayfish, rice crayfish, and wild crayfish using stable isotopes and multi-element data. Although the study successfully achieved its objectives, there are still limitations. For instance, the feed used, tissue types, and season of sample collection could all potentially affect the multi-element and stable isotope profiles of the samples, and the sample size was limited. Further research should not only increase the sample size but also focus on the multi-element and stable isotope characteristics in different tissue parts, determine how these factors influence the model, and seek more stable correlation factors. Additionally, incorporating powerful data processing methods could address these limitations in future studies, enhancing the model’s accuracy and making it an effective tool against crayfish fraud.

## Figures and Tables

**Table 1:** The average values and ANOVA results of stable isotope ratios of 88 crayfish samples from three different farming patterns, including rice crayfish (RC), pond crayfish (PC), and wild crayfish (WC).

Patterns	δ^13^C (‰)	δ^15^N (‰)	δ^2^H (‰)	δ^18^O (‰)
Rice crayfish	−15.58 ± 0.64 ^a^	4.69 ± 0.35 ^bc^	−38.29 ± 3.28 ^a^	−11.40 ± 0.46 ^a^
Pond crayfish	−15.42 ± 0.76 ^a^	5.59 ± 0.18 ^a^	−42.74 ± 2.08 ^b^	−11.66 ± 0.41 ^a^
Wild crayfish	−16.57 ± 0.60 ^b^	5.33 ± 0.32 ^b^	−40.39 ± 4.22 ^bc^	−11.50 ± 0.48 ^a^
*p*-value	0.00	0.00	0.00	0.09

Note: Different lowercase superscripts in the table represent a significant difference between groups at a confidence level of 0.05 (*p* < 0.05).

**Table 2:** The multi-element characteristics of crayfish under collected samples from different farming patterns.

Multi-Element	Rice Crayfish (mg kg^−1^)	Pond Crayfish (mg kg^−1^)	Wild Crayfish (mg kg^−1^)
Be	163.36 ± 67.79	175.01 ± 88.98	179.80 ± 92.25
B	0.17 ± 0.19	0.14 ± 0.17	0.13 ± 0.12
Sc	0.05 ± 0.07	0.04 ± 0.05	0.03 ± 0.03
V	0.08 ± 0.04	0.09 ± 0.04	0.09 ± 0.04
Cr	0.34 ± 0.10	0.35 ± 0.09	0.33 ± 0.08
Mn	20.61 ± 13.61	11.51 ± 4.90	16.32 ± 8.94
Fe	47.01 ± 13.95	49.45 ± 15.77	49.61 ± 13.15
Co	0.07 ± 0.02	0.06 ± 0.02	0.07 ± 0.02
Ni	0.18 ± 0.06	0.20 ± 0.09	0.18 ± 0.07
Cu	13.25 ± 4.39	13.65 ± 4.71	16.03 ± 6.25
Zn	43.46 ± 4.04	41.24 ± 4.89	41.63 ± 4.14
As	0.33 ± 0.13	0.38 ± 0.16	0.39 ± 0.14
Se	0.80 ± 0.13	0.72 ± 0.14	0.80 ± 0.13
Mo	0.32 ± 0.07	0.30 ± 0.08	0.33 ± 0.07
Cd	Nd	Nd	Nd
In	0.00 ± 0.02	Nd	Nd
Sb	0.04 ± 0.02	0.04 ± 0.04	0.04 ± 0.02
Te	0.02 ± 0.01	0.01 ± 0.01	0.02 ± 0.01
Pb	0.06 ± 0.04	0.07 ± 0.04	0.06 ± 0.03
Bi	0.06 ± 0.02	0.06 ± 0.02	0.06 ± 0.03

Note: The LOD of multiple element is 0.01 mg kg^−1^; Nd: no detection.

**Table 3:** Discriminant accuracies of pond crayfish, rice crayfish, and wild crayfish using PLS-DA in the test set.

Processing Method	Farming-Patterns	Number (*n*)	Accuracy (%)
PLS-DA	Pond-crayfish	*n* = 7	89.3%
	Rice-crayfish	*n* = 11	96.4%
	Wild-crayfish	*n* = 10	85.7%
Total accuracy (%)	/	/	90.8%

## Data Availability

The original data presented in the study are included in the article/Supplementary Material, further inquiries can be directed to the corresponding author.

## Supplementary Materials

The following supporting information can be downloaded at: https://www.mdpi.com/article/10.3390/foods13182947/s1, Table S1: Region information of crayfish samples, Table S2: Stable isotope ratio values of crayfish’s feed and environmental samples, Table S3: Multi-element contents of crayfish’s feed and environmental samples.
