# Supplementary material for: Differentiating Pond-Intensive, Paddy-Ecologically, and Free-Range Cultured Crayfish (Procambarus clarkii) Using Stable Isotope and Multi-Element Analysis Coupled with Chemometrics
Source: Foods. 2024 Sep 18;13(18):2947. doi: 10.3390/foods13182947 (PMC11431733; doi:10.3390/foods13182947)
Supplement: Supplementary file 1 [file foods-13-02947-s001.zip › foods-3173044-supplementary.pdf]

Table Captions

TableS1. Region information of crayfish samples.

TableS2. Stable isotope ratio values of crayfish's feed and environmental samples.

TableS3. Multi-element contents of crayfish's feed and environmental samples.

TableS1. Region information of crayfish samples.

Crayfish Type	Crayfish samples	Site	Production method
Pond-crayfish (PC) (*N = 26*)	S1-S5; S9-S13; S19-S23;<br>/<br>S6-S8; S14-S18; S24-S26	Xianning city<br>E'zhou city'<br>Jingzhou city	rice-crayfish co-culture
Rice-crayfish (RC) (*N = 38*)	S27-S31; S42-S44; S57-S61;<br>S32-S36; S48-S54;<br>S37-S41; S45-S47; S55-S56; S62-S64	Xianning city<br>E'zhou city'<br>Jingzhou city	cultured in intensive-pond
Wild-crayfish (WC) (*N = 24*)	S65; S70-S74; S81-S85;<br>S66; S78;<br>S66-S69; S75-S77; S79-S80; S86-S88	Xianning city<br>E'zhou city'<br>Jingzhou city	catched in wild river

TableS2. Stable isotope ratio values of crayfish's feed and environmental samples.

Sample Type	$\delta^{13}C$ (‰)	$\delta^{15}N$ (‰)
Rice (*N = 3*)	-28.94 ± 0.35	4.31 ± 0.76
Soya bean (*N = 4*)	-27.83 ± 0.25	5.15 ± 0.42
Commercial feed (*N = 6*)	-27.96 ± 0.50	6.43 ± 0.63
Aquatic plant (*N = 8*)	-28.63 ± 0.45	3.07 ± 0.58
Sediment (*N = 23*)	-27.33 ± 0.22	2.49 ± 0.18

TableS3. Multi-element contents of crayfish's feed and environmental samples.

Multi-element	Soya bean (mg kg^{-1})	Commercial feed (mg kg^{-1})
Be	20.18	107.26
B	0.05	0.18
Sc	Nd	0.05
V	0.01	0.06
Cr	0.07	0.18
Mn	3.48	10.84
Fe	11.39	34.41
Co	0.02	0.05
Ni	0.04	0.08
Cu	2.08	9.91
Zn	10.64	26.08

As	0.07	0.18
Se	0.17	0.42
Mo	0.08	0.14
Cd	Nd	Nd
In	Nd	Nd
Sb	0.06	0.01
Te	Nd	Nd
Pb	0.02	0.05
Bi	0.02	0.05

Note: the LOD of multi-elements are 0.01 mg kg^{-1}.
